# Effects of Tabata High-Intensity Interval Training on Physiological and Psychological Outcomes in Contemporary Dancers and Sedentary Individuals: A Quasi-Experimental Pre–Post Study

**DOI:** 10.3390/jfmk10040424

**Published:** 2025-11-01

**Authors:** Andrea Francés, Sebastián Gómez-Lozano, Salvador Romero-Arenas, Aarón Manzanares, Carmen Daniela Quero-Calero

**Affiliations:** 1 Facultad de Deporte, UCAM—Universidad Católica de Murcia, Guadalupe, 30107 Murcia, Spain; afrances0@alu.ucam.edu (A.F.); sromero@ucam.edu (S.R.-A.); amanzanares@ucam.edu (A.M.); 2 Performing Arts Research Group, Faculty of Sport, San Antonio Catholic University, 30107 Murcia, Spain; 3 International Chair of Sport Medicine, UCAM Catholic University of Murcia, 30007 Murcia, Spain

**Keywords:** dancers, HIIT, mental health, physical fitness, Sedentarism, Tabata training

## Abstract

**Objectives:** The present study analyzes the effects of a high-intensity interval training (HIIT) program based on the Tabata method on physiological and psychological variables in contemporary dancers (n = 10) and sedentary individuals (n = 8), who performed a 10-week protocol, with sessions of self-loading exercises structured in intervals of 20 s of effort and 10 s of rest three times a week. **Methods:** Parameters of body composition, muscle strength, aerobic and anaerobic capacity, heart rate variability, as well as perceptions of health, anxiety, stress, sleep quality, and levels of physical activity and sedentary lifestyle were evaluated. **Results:** The results showed that no significant changes occurred in most body composition variables, except for visceral fat, where group differences were observed (F = 5.66, *p* = 0.030, *η*^2^_p_ = 0.261). In the indicators of strength and power, the dancers improved the height and relative power of the jump (F = 5.996, *p* = 0.026, *η*^2^_p_ = 0.273), while the sedentary ones increased the strength of the handgrip (*p* = 0.023). In terms of functional performance, both groups significantly increased anaerobic endurance (F = 10.374, *p* = 0.005, *η*^2^_p_ = 0.393), although no changes were recorded in maximal oxygen consumption or heart rate variability (*p* > 0.05). On a psychological level, improvements in healthy lifestyle habits and a decrease in the trait anxiety variable were evidenced in dancers (*p* = 0.023), while in sedentary participants no relevant effects were found. **Conclusions:** In conclusion, the Tabata protocol may represent an efficient and complementary strategy to enhance strength, anaerobic power, and psychological well-being, particularly among dancers. The observed improvements suggest potential benefits related to movement quality, injury prevention, and general physical conditioning.

## 1. Introduction

Contemporary dance is an artistic discipline that imposes high physiological demands, requiring performers to develop and maintain a high level of physical fitness along with mastery of highly specialized technical skills [1]. Physical qualities that are cvdeterminants of optimal performance include cardiorespiratory endurance, muscle strength, anaerobic power, flexibility, motor coordination, and neuromuscular control [2,3]. These abilities not only have a direct impact on the efficiency and expressiveness of movement, but also play a crucial role in injury prevention, regulation of physiological load during stage performance, and long-term sustainability of professional careers [4].

In this context, the incorporation of systematic physical training methods is essential for the comprehensive preparation of dancers [5]. One of the forms of training with multiple benefits in various sports disciplines is high-intensity interval training (HIIT), and, among its variants, the Tabata method, which has shown significant effects on improving maximal oxygen consumption (VO_2_max), anaerobic endurance, neuromuscular strength and recovery capacity [6]. This method is organized into intervals of 20 s of exercise at maximum intensity, followed by 10 s of passive recovery, repeated continuously for a total of four minutes. Its brevity, together with its high effectiveness, has favored its implementation as a training tool adaptable to different populations and levels of physical fitness [7], becoming a particularly useful strategy in intensive trial contexts or with time restrictions, favoring the reduction of fatigue and optimizing recovery processes [8].

In addition, it has been observed that high-intensity interval training contributes to the strengthening of mental health, by reducing levels of anxiety, depression, and tension, promoting increased self-esteem, and optimizing cognitive functions such as attention and working memory [9]. These benefits, linked to the release of endorphins and the stimulation of neuroplasticity, enhance emotional resilience and the ability to adapt to physical and mental demands, aspects of special relevance in the field of dance, where emotional control, concentration and mind–body integration are decisive for artistic performance [10].

Despite growing evidence of the benefits of HIIT in dance [11,12,13], two gaps remain. First, there is a lack of direct, parallel comparisons between contemporary dancers and sedentary adults exposed to an identical Tabata-based HIIT protocol, limiting inference about differential adaptations attributable to prior training rather than differences in protocol. Second, previous studies have rarely integrated physiological and psychological variables, including heart rate variability alongside anxiety, stress, sleep quality, perceived health, and physical activity behavior, within the same intervention, reducing ecological interpretation for performing arts contexts.

Therefore, the aim of this study was to analyze the effects of a 10-week Tabata-based HIIT program on contemporary dancers and sedentary individuals, using a single standardized, supervised, bodyweight-based protocol and a set of multidomain outcomes (body composition, muscle strength/endurance, aerobic and anaerobic capacity, heart rate variability, and psychological measures). Our contribution is to provide a homogeneous comparison of the differential adaptations between trained and untrained cohorts under the same HIIT exposure, jointly characterizing physical and psychological responses. We hypothesized that (1) both groups would improve on several outcomes, (2) relative gains would be greater in sedentary participants due to lower initial fitness, and (3) both cohorts would show favorable changes in heart rate variability (HRV) and psychological measures, supporting the feasibility and translational value of brief Tabata sessions in dance settings with limited schedules.

## 2. Materials and Methods

### 2.1. Study Design

A quasi-experimental study with a pre–post design was conducted over a period of 10 weeks, in which participants completed a supervised HIIT program based on the Tabata protocol [6]. Baseline and post-intervention assessments were administered under identical conditions, using the same instruments and standardized procedures detailed in Section 2.3. To minimize diurnal variability, all measurements were obtained by the same qualified assessor and at the same time of day (morning). Before data collection, participants attended a familiarization session to ensure correct understanding and execution of all testing procedures.

### 2.2. Participants

A total of 21 participants (age: 27.8 ± 7.2 years; height: 166.3 ± 9.1 cm) began the study, of which three of them were excluded from the final analysis for not having completed at least 80% of the scheduled training sessions. Finally, the sample consisted of two mixed groups: dancers (DA; n = 10, 8 females and 2 males) and sedentary participants (SE; n = 8, 6 females and 2 males). The inclusion criteria for the group of dancers were (a) have at least two years of experience in contemporary dance in a conservatory or recognized school; and (b) participate regularly in dance training sessions. For the sedentary group, the criteria were: (a) not to perform any activity related to dance; (b) not participate in any structured physical exercise program for at least 6 months prior to the study and (c) have a sedentary office job. During the evaluation period, any participant who showed signs of illness, injury, or who was under pharmacological treatment was excluded. Detailed information on the objectives and potential risks of the study was provided, and written informed consent was obtained from participants in advance. All experimental procedures were approved by the Ethics Committee of the Universidad Católica San Antonio de Murcia (code CE092201) and were carried out in accordance with the principles of the Declaration of Helsinki.

### 2.3. Procedures

The Tabata protocol was characterized by a work-to-rest ratio of 2:1, consisting of 20 s of exercise at maximum intensity followed by 10 s of rest, grouped into 4-min series. Between sets, one minute of complete recovery was established. The training consisted of self-loading exercises (use of body weight), performed with maximum execution speed maintaining the correct technique three days a week. All participants, both dancers and sedentary groups, carried out the same intervention. The eight exercises included in each session and their order were as follows: squats, push-ups, jumping jacks, front plank, lunge, bench triceps, burpees and abdominal crunch.

All sessions were directly supervised by a certified trainer who provided standardized verbal cues to sustain “maximal effort” corresponding to 9–10 on the Borg CR10 scale during each 20 s of work interval, while ensuring technical execution.

**Figure 1:**
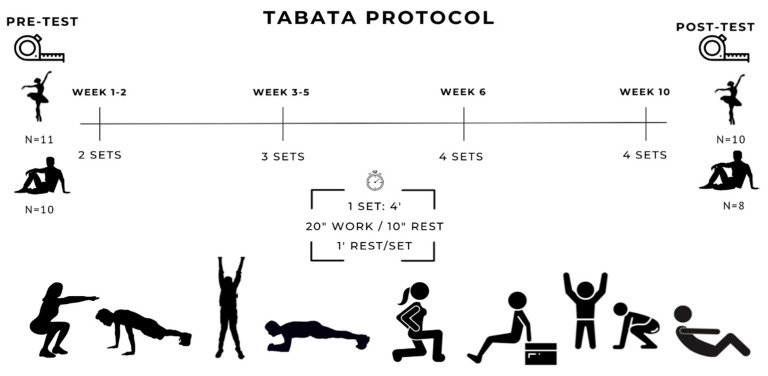
Outline of the experimental design and the 10-week intervention protocol based on the Tabata training method in dancers and sedentary individuals.

The structure of the training sessions remained constant in terms of the type of exercises and their order, all of them being supervised by qualified personnel. Each session began with a warm-up based on joint mobility exercises. Next, the main part was carried out, consisting of four-minute series made up of the eight selected exercises. Throughout the program, the number of sets per session progressively increased from two to four. The session ended with a cool-down, consisting of passive and active static stretches aimed at the main muscle groups involved in the training. Figure 1 shows a diagram of the experimental design and the intervention protocol.

**Figure 2:**
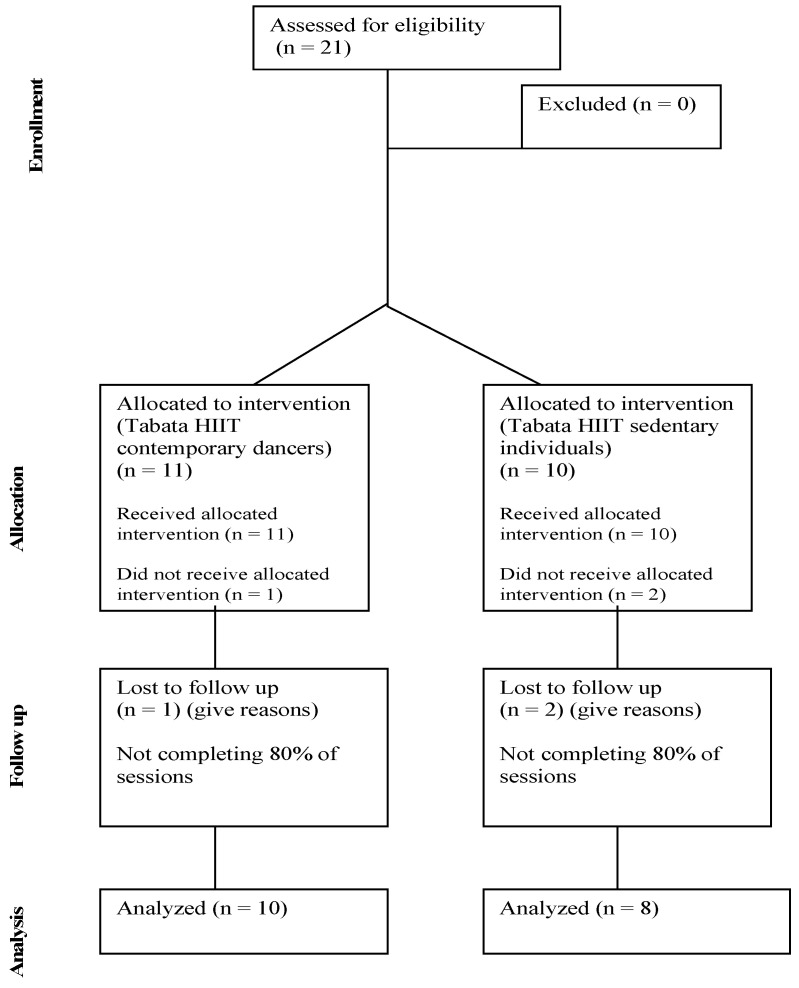
Adapted CONSORT flow diagram for the quasi-experimental Pre–Post study.

A summary of the participant recruitment, allocation, follow-up, and analysis process is presented in Figure 2, following an adapted CONSORT flow diagram for quasi-experimental designs.

#### 2.3.1. Body Composition Testing

As a method of body composition assessment, a TANITA DC-360 digital bioelectrical impedance scale (Tanita, Tokyo, Japan) was used, which allowed the following variables to be recorded: body weight (kg), body mass index (BMI; kg/m^2^), percentage of fat mass (%), percentage of total body water (%), muscle mass (kg), visceral fat (index), bone mass (kg) and basal metabolism (kcal/day). The measurements were carried out at the same time in the pre- and post-period, with the participants fasting and ensuring that they had not consumed caffeine or alcohol for at least 24 h prior to the evaluation. Participants had to be barefoot, wearing light clothing and no metal objects (jewelry, watches) during the measurement, to avoid interference with the analysis.

#### 2.3.2. Strength Testing

Upper limb hand grip strength was assessed using the TKK 5105 dynamometer (Takei Scientific Instruments Co., Ltd., Niigata, Japan). Participants performed the test in an upright position, with their arms extended next to their body, avoiding contact with the trunk and keeping their shoulders in neutral adduction and rotation. Three attempts were made for each hand (dominant first, followed by non-dominant), with the highest value achieved in each of the members being recorded as the final result

The counter movement jump (CMJ) was evaluated to measure the power of the lower limb. The test was carried out on a MuscleLab contact platform (Ergotest Technology, Langesund, Norway). The participants began in an upright position, with their hands on their waists to avoid arm thrust, and performed a vertical jump preceded by a rapid bending of the knees (eccentric phase) followed by an explosive extension (concentric phase), without pausing between the two phases. Three attempts were made with a rest time of 30 s between them, and the highest jump was recorded as the final result.

#### 2.3.3. Perceived Levels of Physical Activity, Overall Health, Stress, Anxiety and Sleep Quality

Previously validated questionnaires were used for the subjective determination of the levels of general health, physical activity, stress, anxiety and sleep quality.

To measure physical activity, the international physical activity questionnaire (IPAQ) was used in its extensive Spanish version [14]. This instrument allowed estimating the weekly energy expenditure in MET-min/week, considering levels of vigorous, moderate and light activity, as well as the daily sedentary time. It was used before and after the training program to analyze changes in physical activity patterns.

Healthy Lifestyle and Personal Control Questionnaire: The Healthy Lifestyle and Personal Control Questionnaire (HLPCQ) is an instrument designed to identify and quantify lifestyle patterns related to health empowerment, personal control and perceived stress. It assesses dimensions such as healthy and harmful diet, routine, physical exercise, and mental health [15].

Trait and State Anxiety Questionnaire (STAI) designed to assess participants’ level of anxiety. It is divided into two sections: trait anxiety, which measures how they usually feel, and state anxiety, which reflects how they feel at a particular time. On both scales, higher scores indicate a higher level of anxiety [16].

The Perceived Stress Scale (PSS) is a questionnaire designed to assess how often participants feel that demand exceeds their ability to cope with them, considering aspects such as unpredictability, lack of control and overload. The higher the score, the higher the level of perceived stress [17].

Sleep quality questionnaire, through a questionnaire (Karolinska Sleep Diary, KSD) validated in the adult population that allows subjectively assessing sleep quality in different dimensions, such as daytime sleepiness, subjective perception of sleep quality, the presence of nocturnal awakenings and difficulties in maintaining sleep. Higher scores reflect poorer quality in the aspect assessed [18].

#### 2.3.4. Aerobic and Anaerobic Testing

To assess the participants’ anaerobic endurance, the 3-min burpees test was used, a high-intensity functional test designed to estimate the ability to sustain repeated efforts of short duration. The protocol consisted of performing as many complete burpees as possible during a continuous period of three minutes. Each burpee included a squat, hand rest, back leg extension, return to the squat position, and a vertical jump. The number of repetitions performed in each minute was recorded, as well as the cumulative total at the end of the test. The repetitions were supervised to ensure correct technical execution, discarding those that did not meet the established criteria.

To assess the aerobic endurance of the participants, the Course Navette test or 20-m round-trip test was used, a widely validated protocol to estimate maximum oxygen consumption (VO_2_max) indirectly. The test consisted of running between two lines separated by 20 m following the rhythm marked by an audible signal that progressively increases speed. Participants had to adjust their running to the rhythm of the audio, increasing the intensity as the levels progressed. The race ended when the participant failed to reach the finish line on two consecutive occasions at the required pace. The last completed stage was recorded, which was later used to estimate VO_2_max through validated formulas [19] in its absolute and relative value.

#### 2.3.5. Heart Rate Variability (HRV) Testing

To assess the cardiovascular health of the participants, Heart Rate Variability (HRV) was used as the main indicator. The measurements were performed in the early morning, using a Polar H7 heart rate monitor (Kempele, Finland), secured with a chest strap below the sternum. Participants remained in a supine position in a quiet environment for a 5-min recording period, preceded by a 5-min stabilization phase to ensure resting heart rate conditions. Subsequently, the analysis of HRV-related variables was carried out using the specialized Kubios HRV 3.0 software (Kuopio, Finland). Parameters such as mean R-R intervals (RR), standard deviation of consecutive intervals (SDNN), mean square root of differences between consecutive R-R intervals (RMSSD), percentage of intervals differing by more than 50 milliseconds (pNN50), mean heart rate (Mean HR), high-frequency power (HF), and ratio of low-frequency to high-frequency power (LF/HF) were recorded. Where necessary, very low, low or medium threshold corrective filters were applied using the same software to adjust for possible artifacts.

### 2.4. Statistical Analysis

The statistical analysis was performed using Jamovi software (version 2.3.28). A two-way repeated measures analysis of variance was applied to evaluate the effects of high-intensity interval training on the different variables, considering an intra-subject factor (time: pre vs. post intervention) and an inter-subject factor (group: dancers vs. sedentary). Previously, the assumptions of normality for each variable were verified using the Shapiro–Wilk test. In all cases, the data met the normality criterion (*p* > 0.05), so parametric analysis was performed. The main effects of time, the group and its interaction (time × group) were examined. Statistical significance was established at *p* < 0.05. In addition, the effect size was reported by partial eta square (*η*^2^_p_), considering values of 0.01, 0.06 and 0.14 as small, medium and large, respectively.

## 3. Results

After a period of 10 weeks of Tabata training, no statistically significant changes were observed in most of the body composition variables analyzed (Table 1), including body weight, body mass index (BMI), fat mass, total body water, muscle mass, bone mass, and basal metabolism, with no significant effects of time, of the group or the interaction between the two (all values of *p* > 0.05). The only significant difference was found in visceral fat, with a group effect (F = 5.66, *p* = 0.030, *η*^2^_p_ = 0.261), probably attributable to initial differences between the group of dancers and sedentary individuals.

Specific improvements in strength and power were identified (Table 2). The sedentary group significantly improved the manual grip strength of the non-dominant hand (*p* = 0.023) and showed a tendency to improve in the dominant hand (*p* = 0.058). On the other hand, the group of dancers significantly increased the height of the jump (CMJ) (*p* = 0.033). Likewise, a significant interaction was found in the relative power of the jump (F = 5.996, *p* = 0.026, *η*^2^_p_ = 0.273), which reflected opposite responses between the groups: the dancers increased their power, while the sedentary individuals reduced it.

The subjective results regarding physical activity and sedentary behavior are presented in Table 3 and reflect a significant increase in the overall level of physical activity, especially in the group of dancers, which showed a significant increase in total weekly energy expenditure (*p* = 0.040), with an equally significant time effect (F = 10.21, *p* = 0.006, *η*^2^_p_ = 0.390). Although no significant changes were found in vigorous, moderate, or light activity, improvement trends were observed in these variables within the group of dancers.

In reference to the dimensions of healthy lifestyle and in some aspects of mental health (Table 4). There were positive effects on daily routine, perceived physical exercise, and total healthy lifestyle score, reflecting widespread improvements after the intervention. Trait anxiety decreased in dancers (F = 6.30, *p* = 0.023, *η*^2^_p_ = 0.283), while it increased slightly in sedentary participants. Although trends of improvement in diet were observed in the dancers, they did not reach statistical significance. No changes were found in mental health, sleep, perceived stress or state anxiety, indicating that the protocol mainly influenced active behavior and perception of control.

The cardiorespiratory condition in both groups is reflected in Table 5, mainly evidenced by the total number of burpees performed (F = 10.374, *p* = 0.005, *η*^2^_p_ = 0.393), as well as in those completed in each of the minutes, which reflects a significant effect of time on muscular and anaerobic endurance. No group or interaction effects were detected (*p* > 0.05), suggesting that both dancers and sedentary individuals improved equally. In contrast, there were no significant changes in maximal heart rate or maximal oxygen consumption (VO_2_ max, relative and absolute). Likewise, no significant changes were observed in any of the indicators of heart rate variability (HRV) in dancers or sedentary individuals (Table 6). Variables such as RR, SDNN, RMSSD, pNN50, absolute HF, and the LF/HF ratio showed no time, group, or interaction effects (*p* > 0.05), indicating that the intervention did not produce adaptations in cardiac autonomic regulation.

## 4. Discussion

The purpose of this research was to compare the effects of a high-intensity protocol based on the Tabata method, carried out over 10 weeks, on cardiorespiratory capacity, body composition, muscle strength, as well as on the mental health and quality of life of contemporary dancers and sedentary subjects. The results obtained allowed the identification of differentiated patterns of adaptation, both in physiological and functional variables and in lifestyle and psychological health indicators.

Tabata training, as a form of high-intensity interval training, activates metabolic processes through high-intensity exercise, promoting the elimination of lactate and H^+^ (hydrogen ions), glycogen resynthesis and increased growth hormone levels. These adaptations enhance fat oxidation and weight reduction, as well as improve athletic performance and glucose metabolism [20]. According to our results, no statistically significant changes in overall body composition were observed, consistent with previous studies reporting limited effects of short-duration HIIT training on variables such as body weight, body mass index (BMI), or fat mass in active individuals or individuals with a low body fat percentage [21] as observed in dancers. However, a significant reduction in visceral fat was detected with a differential effect by group, suggesting that the protocol may have generated specific benefits on metabolic health, particularly in participants with higher levels of abdominal adiposity at the beginning of the intervention [22]. Nevertheless, this result should be interpreted with caution, as the observed group effect (F = 5.66, *p* = 0.030, *η*^2^_p_ = 0.261) may be partly explained by baseline differences between dancers and sedentary participants rather than by the intervention itself.

In relation to physical activity patterns and sedentary behavior, dancers experienced a significant increase in weekly energy expenditure (F = 10.21, *p* = 0.006, *η*^2^_p_ = 0.390), as well as positive, though non-significant, tendencies toward greater engagement in vigorous (F = 2.50, *p* = 0.133, *η*^2^_p_ = 0.136) and moderate activities (F = 3.23, *p* = 0.091, *η*^2^_p_ = 0.168). Although no statistically significant changes in total sedentary time were detected (F = 3.17, *p* = 0.094, *η*^2^_p_ = 0.165), the slight reduction observed in both groups could represent a relevant behavioral improvement if maintained over the long term, especially considering the health risks associated with prolonged inactivity [23]. In addition, the amount of vigorous activity among dancers remained limited, while sedentary time was relatively high. This pattern persisted even after the training program, suggesting that despite the high physical demands of dance, the volume of intense activity does not seem to reach the thresholds usually associated with physically trained populations. A study on professional dancers [24] also reported substantial sedentary time throughout the day. These findings, together with our results, suggest that although dance practice is physically demanding at specific times, it does not necessarily ensure a globally active lifestyle, underscoring the need to design complementary interventions that promote sustained physical activity and improve overall fitness levels.

Various meta-analyses have determined that dancing can improve quality of life and reduce symptoms of depression and anxiety, also favoring subjective well-being, positive mood, perceived stress, affection, and body image [25,26]. Regarding our results in the indicators of healthy lifestyle and mental health, the Tabata protocol promoted significant improvements in the organization of the daily routine (F = 6.92, *p* = 0.018, *η*^2^_p_ = 0.289), in the perception of physical exercise (F = 6.43, *p* = 0.021, *η*^2^_p_ = 0.274) and in the overall score of healthy habits (F = 9.15, *p* = 0.008, *η*^2^_p_ = 0.350). In addition, a significant decrease in trait anxiety levels (F = 5.32, *p* = 0.034, *η*^2^_p_ = 0.238) was observed in the group of dancers, which could be linked to the expressive and self-regulating value of movement in artistic contexts, as well as to the perceived improvement in body and emotional control [27]. Although no significant effects were identified on other dimensions such as sleep quality (F = 1.24, *p* = 0.282, *η*^2^_p_ = 0.068) or state anxiety (F = 2.05, *p* = 0.169, *η*^2^_p_ = 0.108), the results point towards an overall positive impact on the psychological well-being of both groups.

In terms of muscle strength and power, differentiated adaptations were observed according to the profile of the participants. In the group of sedentary subjects, a significant improvement in manual grip strength was recorded (F = 6.57, *p* = 0.020, *η*^2^_p_ = 0.279), while dancers showed significant increases (F = 8.47, *p* = 0.010, *η*^2^_p_ = 0.333) in vertical jump (CMJ) and relative power (F = 6.83, *p* = 0.018, *η*^2^_p_ = 0.286). These findings reflect a positive effect of the Tabata protocol on neuromuscular capacity, modulated by the level of initial physical condition and by the type of stimulus predominant in each group, which is consistent with what has been described in research on specific training [2,3]. Recent literature has highlighted the positive effects of different strength training protocols on young dancers [28,29] pointing out that both plyometric programs and those focused on strengthening the muscles, especially of the lower limbs, are effective in optimizing muscle power [30] facilitating the development of faster and more efficient motor unit activation patterns, which are essential for the production of force and maximum power.

Several studies affirm that the incorporation of HIIT as a complementary component in dance training programs can be particularly beneficial to improve aerobic capacity in dancers from various disciplines [3,11]. In addition, the implementation of Tabata training significantly improved performance indicators such as sprint speed, muscle strength, and explosive power, suggesting relevant improvements in the anaerobic condition of college dancers [12]. This coincides with the results of our research, where relevant functional improvements were evidenced, reflected in the increase in the total number of burpees performed (F = 7.54, *p* = 0.015, *η*^2^_p_ = 0.307) and sustained performance for one, two and three minutes. These improvements, common to both groups, indicate an increase in exercise tolerance and anaerobic muscle endurance, in line with what has been reported in HIIT programs [31,32].

Beyond the observed enhancements in anaerobic performance, the potential impact of HIIT on aerobic capacity and HRV has also been highlighted in dancers in previous research [3,12]. Experimental interventions have shown that adjusting exercise intensity based on heart rate variability can enhance the efficiency of physical conditioning in dancers, improving cardiovascular capacity and recovery [33,34]. Similarly, it has been reported that integrating HIIT into dance training programs as supplementary exercise may effectively improve aerobic capacity in female flamenco dancers [11]. However, in the present study, no significant changes were observed in maximal oxygen consumption (F = 1.19, *p* = 0.290, *η*^2^_p_ = 0.065), maximal heart rate (F = 0.84, *p* = 0.372, *η*^2^_p_ = 0.047), or HRV indices, suggesting that the duration of the intervention may not have been sufficient to elicit measurable adaptations in cardiac autonomic regulation.

Future studies should consider including a larger sample size and incorporating control groups consisting of both dancers and sedentary individuals. In addition, dietary monitoring should be considered, as nutritional factors may influence physical performance and mental state. A longitudinal follow-up of at least 6–12 months will also be relevant to assess the durability of the observed effects and the associated injury rates, as well as the use of direct assessment methods such as cardiopulmonary exercise testing (CPET) to obtain more accurate and comprehensive physiological data.

## 5. Conclusions

The present study suggests that a 10-week Tabata-based program may represent a feasible and accessible strategy to improve certain strength, anaerobic endurance, and psychological well-being parameters, with a tendency toward greater effects in dancers compared with sedentary participants. Although no significant changes were observed in most cardiorespiratory indicators, some adaptations observed could potentially contribute to enhanced artistic performance, reduced injury risk, and the promotion of healthy lifestyle habits. These results support the incorporation of the Tabata method as a complement within dance fitness programs, while pointing to the need for longitudinal studies of longer duration to assess its impact on central physiological variables such as VO_2_max or heart rate variability.

## Figures and Tables

**Table 1:** Body Composition Outcomes in Dancers and Sedentary Individuals After 10 Weeks of High-Intensity Interval Training (TABATA).

							ANOVA (F, *p*, η^2^_p_)								
Outcome	Group DA (n = 10); SE (n = 8)	PRE-Training		POST-Training		*p*	Time Effect			Group Effect			Time × Group Effect		
		M	SD	M	SD		F	*p*	η^2^_p_	F	*p*	η^2^_p_	F	*p*	η^2^_p_
Body Weight (kg)	DA	61.3	10.7	62.6	12.1	0.212	1.732	0.207	0.098	1.26	0.279	0.073	0.175	0.681	0.011
	SE	67.6	11.3	68.2	10.9	0.556									
BMI (kg/m^2^)	DA	22.3	2.02	22.6	2.40	0.322	2.989	0.103	0.157	1.22	0.286	0.071	0.134	0.719	0.008
	SE	23.7	3.41	24.1	3.31	0.285									
Fat Mass (%)	DA	24.4	6.19	24.7	6.01	0.743	0.164	0.691	0.010	<0.001	0.993	0.000	<0.001	0.968	0.000
	SE	24.5	5.50	24.7	6.76	0.81									
Body water (%)	DA	55.6	4.11	55.5	3.60	0.89	0.039	0.845	0.002	0.370	0.552	0.023	<0.001	0.990	0.000
	SE	54.6	3.31	54.5	4.11	0.889									
Muscle Mass (kg)	DA	43.8	9.80	44.3	9.49	0.326	1.485	0.241	0.085	1.03	0.326	0.060	0.017	0.897	0.001
	SE	48.6	9.95	49.0	10.4	0.476									
Visceral Fat (index)	DA	2.15	1.25	2.25	1.36	0.601	0.017	0.896	0.001	5.66	**0.030**	0.261	0.334	0.571	0.020
	SE	4.31 *	2.48	4.25 *	2.33	0.769									
Bone Mass (kg)	DA	2.35	0.499	2.35	0.477	0.204	0.593	0.453	0.036	1.5	0.300	0.067	0.593	0.453	0.036
	SE	2.59	0.476	2.61	0.522	0.988									
Basal Metabolism (kcal/day)	DA	1411	274	1404	270	0.566	0.283	0.602	0.017	0.972	0.339	0.057	0.062	0.806	0.004
	SE	1539	274	1536	296	0.852									

* *p* < 0.05 compared to DA; DA: dancers; SE: sedentary; BMI: Body Mass Index; bold: to highlight the statistical results.

**Table 2:** Strength Outcomes in Dancers and Sedentary Individuals After 10 Weeks of High-Intensity Interval Training (TABATA).

							ANOVA (F, *p*, η^2^_p_)								
Outcome	Group DA (n = 10); SE (n = 8)	PRE-Training		POST-Training		*p*	Time Effect			Group Effect			Time × Group Effect		
		M	SD	M	SD		F	*p*	η^2^_p_	F	*p*	η^2^_p_	F	*p*	η^2^_p_
Handgrip dominant (kg)	DA	27.9	7.53	27.9	7.04	0.984	3.51	0.080	0.190	2.50	0.135	0.143	3.41	0.085	0.185
	SE	32.0	8.55	37.1 *	10.1	0.058									
Handgrip non-dominant (kg)	DA	26.4	7.16	26.6	7.30	0.803	4.15	0.059	0.206	1.53	0.233	0.088	2.89	0.109	0.153
	SE	29.9	8.14	32.5	10.1	0.023									
Height CMJ (cm)	DA	19.5	5.32	22.4	4.17	0.033	1.94	0.183	0.108	0.330	0.574	0.020	2.98	0.109	0.157
	SE	22.7	7.71	22.4	7.20	0.826									
Power CMJ (W/kg)	DA	11.7	1.75	12.9	2.95	0.237	0.657	0.430	0.039	0.297	0.593	0.018	5.996	**0.026**	0.273
	SE	14.1	4.41	11.8	1.96	0.044									

* *p* < 0.05 compared to DA, DA: dancers; SE: sedentary; CMJ: Countermovement Jump; bold: to highlight the statistical results.

**Table 3:** Physical Activity Parameters in Dancers and Sedentary Individuals After 10 Weeks of High-Intensity Interval Training (TABATA).

							ANOVA (F, *p*, η^2^_p_)								
Outcome	Group DA (n = 10); SE (n = 8)	PRE-Training		POST-Training		*p*	Time Effect			Group Effect			Time × Group Effect		
		M	SD	M	SD		F	*p*	η^2^_p_	F	*p*	η^2^_p_	F	*p*	η^2^_p_
Vigorous activity (MET-min/week)	DA	696	583	1160	1291	0.238	2.5	0.133	0.136	0.057	0.814	0.004	0.002	0.960	0.000
	SE	790	777	1225	834	0.319									
Moderate activity (MET-min/week)	DA	592	583	1172	1409	0.068	3.231	0.091	0.168	3.78	0.070	0.191	0.654	0.430	0.039
	SE	145	191	365	307	0.517									
Light activity (MET-min/week)	DA	856	968	1531	2037	0.071	2.09	0.167	0.116	0.449	0.512	0.027	1.28	0.275	0.074
	SE	792	663	875	545	0.835									
Sedentarism Time (h/day)	DA	5.75	2.66	5.13	3.52	0.108	3.168	0.094	0.165	0.054	0.818	0.003	0.240	0.631	0.015
	SE	6.40	4.97	5.30	3.59	0.4									
METs (min/week)	DA	2102	1272	3863	2433	**0.04**	10.21	**0.006**	0.390	1.74	0.206	0.98	1.70	0.211	0.96
	SE	1723	1379	2465	870	0.222									

DA: dancers; SE: sedentary; h: hour; min: minutes; bold: to highlight the statistical results.

**Table 4:** Mental Outcomes in Dancers and Sedentary Individuals After 10 Weeks of High-Intensity Interval Training (TABATA).

							ANOVA (F, *p*, η^2^_p_)								
Outcomes	Group DA (n = 10); SE (n = 8)	PRE-Training		POST-Training		*p*	Time Effect			Group Effect			Time × Group Effect		
		M	SD	M	SD		F	*p*	η^2^_p_	F	*p*	η^2^_p_	F	*p*	η^2^_p_
Healthy diet	DA	14.0	3.80	16.1	4.31	0.04	4.439	0.051	0.217	0.099	0.757	0.006	0.753	0.398	0.045
	SE	15.1	3.31	16.0	3.25	0.418									
Unhealthy diet	DA	8.20	3.22	9.70	3.33	0.05	3.995	0.063	0.200	0.007	0.931	0.000	0.677	0.423	0.041
	SE	8.75	2.55	9.38	3.13	0.442									
Rutine	DA	19.0	4.81	20.7	3.77	0.098	8.869	**0.009**	0.357	1.19	0.292	0.069	0.406	0.533	0.025
	SE	16.4	4.57	19.1	4.69	0.028									
Physical exercise	DA	6.40	1.71	7.10	1.10	1	7.68	0.014	0.324	20.7	**<0.001**	0.564	1.41	0.252	0.081
	SE	3.75	1.28	5.50	1.20	0.104									
Mental health	DA	10.8	2.70	12.6	2.63	0.045	2.74	0.117	0.146	1.24	0.283	0.072	1.57	0.229	0.089
	SE	12.8	1.67	13.0	3.07	0.79									
Total Healthy Lifestyle	DA	58.4	9.07	66.2	6.49	0.093	19.855	**<0.001**	0.554	0.517	0.482	0.031	0.287	0.599	0.018
	SE	56.8	7.76	62.9	8.51	0.018									
Sleep Quality	DA	7.75	4.30	6.60	2.46	0.093	0.0269	0.872	0.002	1.36	0.261	0.078	3.385	0.084	0.175
	SE	5.00 *	2.07	6.38	2.62	0.189									
PSS	DA	29.8	6.68	26.1	7.53	0.221									
	SE	24.8	7.05	28.6	4.66	0.25	0.001	0.968	0.000	0.308	0.587	0.019	3.020	0.101	0.159
State-Anxiety	DA	26.0	3.50	26.8	5.45	0.555	0.162	0.693	0.010	0.042	0.839	0.003	0.162	0.693	0.010
	SE	26.0	4.07	26.0	5.10	1									
Trait-Anxiety	DA	29.1	4.77	24.3	4.40	**0.007**	2.71	0.120	0.145	2.90	0.108	0.153	6.30	**0.023**	0.283
	SE	22.9 *	2.47	23.9	6.66	0.57									

* *p* < 0.05 compared to DA. Values represent total scores for each outcome measure. DA: dancers; SE: sedentary; PSS: Perceived Stress Scale; bold: to highlight the statistical results.

**Table 5:** Comparison between dancers and sedentary individuals in cardiorespiratory measures.

							ANOVA (F, *p*, η^2^_p_)								
Outcome	Group DA (n = 10); SE (n = 8)	PRE-Training		POST-Training		*p*	Time Effect			Group Effect			Time × Group Effect		
		M	SD	M	SD		F	*p*	η^2^_p_	F	*p*	η^2^_p_	F	*p*	η^2^_p_
Burpees total (nº)	DA	32.6	8.04	38.1	6.84	0.035	10.374	**0.005**	0.393	0.127	0.726	0.008	0.019	0.890	0.001
	SE	31.3	8.15	37.3	6.96	0.039									
Burpees 1 min (nº)	DA	14.4	2.37	15.6	2.37	0.128	14.00	**0.002**	0.467	<0.001	1.000	0.000	2.57	0.128	0.138
	SE	13.5	3.30	16.5	2.62	**0.02**									
Burpees 2 min (nº)	DA	9.00	3.37	11.7	2.58	0.023	7.670	**0.014**	0.324	0.035	0.853	0.002	0.35	0.563	0.021
	SE	9.25	3.01	11.0	3.16	0.163									
Burpees 3 min (nº)	DA	9.20	3.01	10.8	2.86	0.078	4.983	**0.040**	0.237	0.527	0.478	0.032	0.075	0.787	0.005
	SE	8.50	2.93	9.75	2.66	0.200									
HR max (bpm)	DA	181	5.34	180	14.6	0.825	0.592	0.453	0.036	0.096	0.760	0.006	0.221	0.645	0.014
	SE	182	13.3	175	30.3	0.418									
VO_2_ max relative (mL/kg/min)	DA	36.8	5.22	37.6	3.20	0.769	2.06	0.170	0.114	0.281	0.603	0.017	1.08	0.315	0.063
	SE	33.3	6.87	38.1	11.8	0.117									
VO_2_ max absolute (L/min)	DA	2.25	0.520	2.36	0.618	0.606	2.515	0.132	0.136	0.134	0.719	0.008	0.783	0.389	0.047
	SE	2.22	0.416	2.59	0.878	0.117									

DA: dancers; SE: sedentary; nº: number of burpees; bpm: beats per minute; mL/kg/min: milliliters per kilogram of body weight per minute; L/min: liters per minute; bold: to highlight the statistical results.

**Table 6:** Comparison between dancers and sedentary individuals in Heart Rate Variability measures.

							ANOVA (F, *p*, η^2^_p_)								
Outcome	Group DA (n = 10); SE (n = 8)	PRE-Training		POST-Training		*p*	Time Effect			Group Effect			Time × Group Effect		
		M	SD	M	SD		F	*p*	η^2^_p_	F	*p*	η^2^_p_	F	*p*	η^2^_p_
RR (ms)	DA	1000	129	996	157	0.582	0.049	0.828	0.003	0.279	0.227	0.090	1.58	0.605	0.017
	SE	904	177	918	156	0.84									
SDNN (ms)	DA	75.4	45.9	82	58.8	0.666	0.526	0.479	0.032	0.110	0.744	0.007	0.019	0.891	0.001
	SE	67.4	26.4	77	50.1	0.57									
Mean HR (bpm)	DA	60.9	7.66	63.7	10.6	0.512	0.042	0.840	0.003	1.97	0.179	0.110	0.476	0.500	0.029
	SE	68.5	12.6	67	11.2	0.749									
RMSSD (ms)	DA	71.7	47.9	89.8	89.5	0.373	0.319	0.580	0.020	2.21	0.157	0.121	0.433	0.520	0.026
	SE	47.7	22.7	46.3	34.6	0.951									
pNN50 (%)	DA	36.3	23.6	40	30.2	0.689	0.189	0.670	0.012	3.73	0.071	0.189	0.112	0.915	0.001
	SE	19.2	15.9	21.4	23	0.83									
Hf absolute (ms^2^)	DA	2503	2617	2770	3727	0.755	0.366	0.554	0.022	1.64	0.219	0.093	0.033	0.858	0.002
	SE	974	847	1470	2349	0.605									
LF/HF ratio	DA	1.05	0.98	1.02	1.03	0.946	0.022	0.885	0.001	3.44	0.082	0.177	0.003	0.957	0.000
	SE	1.82	1.26	1.76	1.03	0.894									

DA: dancers; SE: sedentary; RR: mean R–R interval; SDNN: standard deviation of NN intervals; RMSSD: root mean square of successive differences; pNN50: percentage of NN intervals >50; HF: high-frequency power; LF/HF: ratio of low- to high-frequency power. Mean HR: mean heart rate; ms: milliseconds; bpm: beats per minute; %: percentage.

## Data Availability

The original contributions presented in this study are included in the article. Further inquiries can be directed to the corresponding author.

## Abbreviations

The following abbreviations are used in this manuscript:

HIIT	High-Intensity Interval Training
VO_2_max	Maximal Oxygen Consumption
DA	Dancers
SE	Sedentary Individuals
CMJ	Counter Movement Jump
IPAQ	International Physical Activity Questionnaire
HLPCQ	Healthy Lifestyle and Personal Control Questionnaire
STAI	Trait and State Anxiety Questionnaire
PSS	Perceived Stress Scale
KSD	Karolinska Sleep Diary
HRV	Heart Rate Variability
R-R	R-R intervals
SDNN	Standard Deviation of Consecutive Intervals
RMSSD	Mean square root of differences between consecutive R-R intervals
pNN50	Percentage of intervals differing by more than 50 milliseconds
Mean HR	Mean heart rate
HF	High-frequency power
LF/HF	Ratio of low-frequency to high-frequency power
η^2^p	Partial eta square
BMI	Body Mass Index
